# Unusual Occurrence of Tenosynovial Giant Cell Tumor in Hoffa's Fat Pad: A Potential Differential Diagnosis for Nontraumatic Knee Swelling and Pain

**DOI:** 10.7759/cureus.9008

**Published:** 2020-07-05

**Authors:** Rakesh Goyal, Rajat Chopra, Nishant Bhatia, Akash Goel, Palash Gupta

**Affiliations:** 1 Department of Orthopaedics, Sports Injury Centre, Vardhman Mahavir Medical College and Safdarjung Hospital, New Delhi, IND; 2 Department of Orthopaedic, Sir Ganga Ram Hospital, New Delhi, IND; 3 Department of Orthopaedic Surgery, Maulana Azad Medical College and Associated Lok Nayak Hospital, New Delhi, IND; 4 Orthopaedics, Maulana Azad Medical College, Delhi, IND

**Keywords:** hoffa's fat pad, knee, tenosynovial giant cell tumor

## Abstract

A tenosynovial giant cell tumor (TGCT) is a benign lesion whose presence in Hoffa's pad has rarely been reported. This unique case report discusses a 33-year-old female patient who presented with swelling and pain in her left knee. Clinical and MRI findings were used to make the diagnosis, which was confirmed on a histopathological basis. The patient had a large tumor (5 × 3 × 3 cm) in Hoffa's fat pad, which was diagnosed as TGCT and managed with open resection due to its size. At follow-up after 20 months, the patient was asymptomatic, and there was no local recurrence of the tumor.

## Introduction

A tenosynovial giant cell tumor (TGCT) is a benign lesion of the tendon sheath, synovial membrane, or bursa [1]. It manifests in two forms, consisting of a localized form that involves an area of tendon sheath or synovial membrane, whereas the diffuse form is extensively involved with the synovium [2,3]. Localized TGCT is usually found in the tendon sheath of the hand or foot. However, its presence in Hoffa's fat pad, which is located underneath the patella, is an unusual finding [4]. Herein, we report a unique case of TGCT in Hoffa's fat pad in a young female patient who was treated with open resection of the mass.

## Case presentation

A 33-year-old female patient presented to the outpatient department with concerns of swelling and pain in her left knee. She first noticed swelling in her left knee three and a half months earlier and was relatively asymptomatic. The swelling was insidious at onset, nonprogressive, and associated with pain. Clinical examination revealed a 5 cm × 3 cm swollen area in the anteromedial aspect of the left knee with ill-defined borders. The overlying skin was intact, and the swelling did not affect the skin or underlying bone. The swollen area was tender on palpation, but with no local increase in temperature.

Clinical tests for cruciate ligaments, collateral ligaments, and menisci did not reveal any abnormalities. The patient's knee was stable without any restriction in the range of motion. There was no distal neurovascular deficit. The Oxford knee score was 32 at the time of presentation [5]. The patient had no previous history of trauma nor any medical comorbidities.

**Figure 1:**
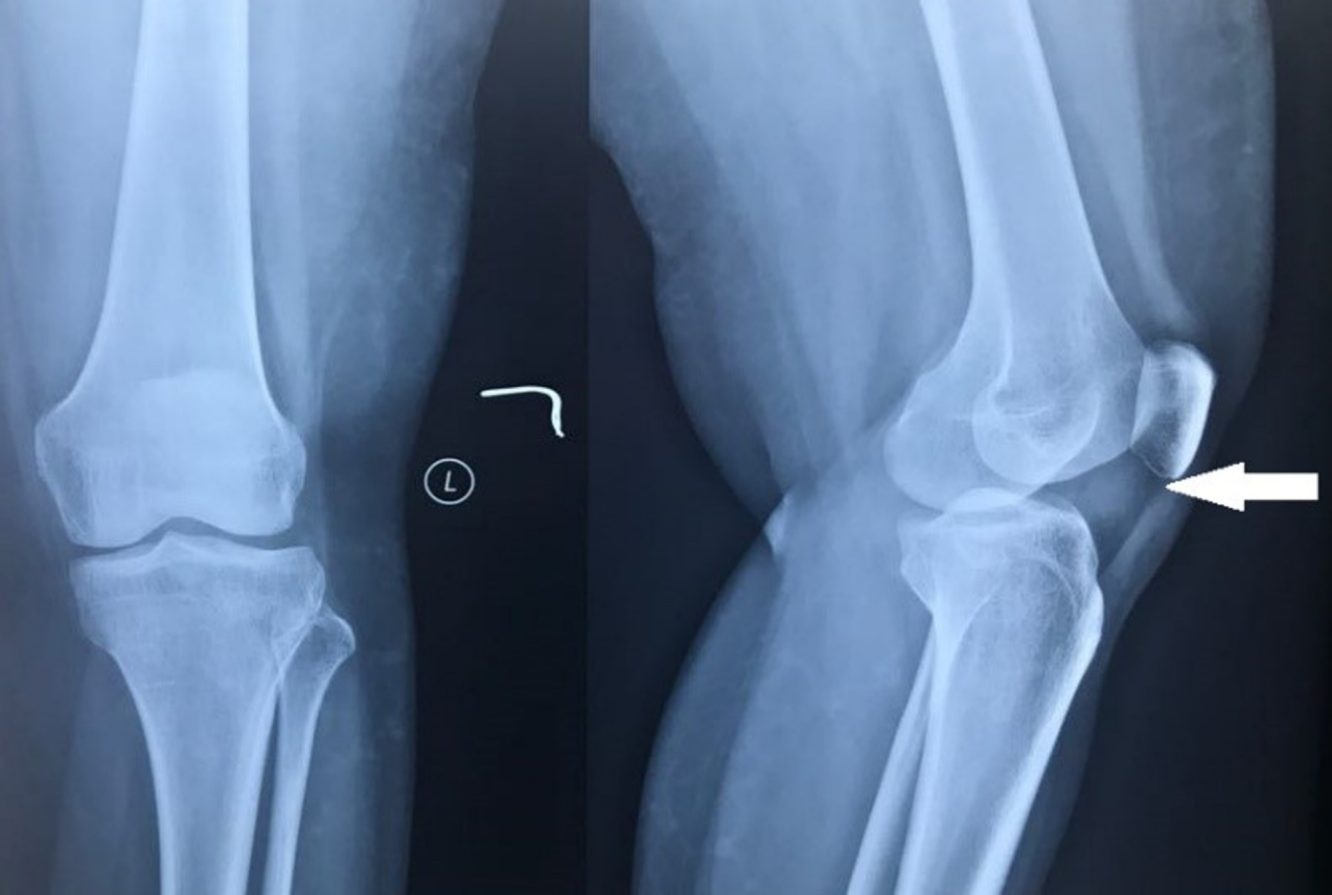
Anteroposterior and lateral radiographs of the knee joint showing opacity in infrapatellar region (white arrow).

Radiological investigations were performed, and standard radiographs of the left knee showed opacity in the infrapatellar region (Figure 1).

**Figure 2:**
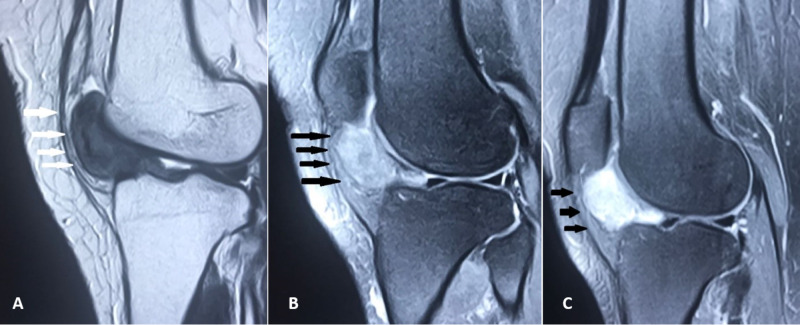
MRI of the left knee. MRI showing (A) isointensity to muscle (white arrows) in T1-weighted images, (B) low to intermediate signal intensity (black arrows) in T2 images, (C) post-contrast homogeneous enhancement (black arrows) in T1-weighted fat-suppressed images.

MRI of the left knee showed a well-marginated soft tissue space occupying lesion in Hoffa's fat pad abutting the anterior joint capsule, with isointensity to muscle in T1-weighted images (Figure 2A) and low to intermediate signal intensity in T2 images (Figure 2B). Intense homogeneous enhancement in the lesion was evident after contrast administration in T1-weighted fat-suppressed images (Figure 2C). MRI findings were suggestive of focal nodular pigmented villonodular synovitis/synovial hemangioma or chondroma. The MRI showed normal intensity and morphology for the cruciate ligaments, collateral ligaments, and menisci. 

**Figure 3:**
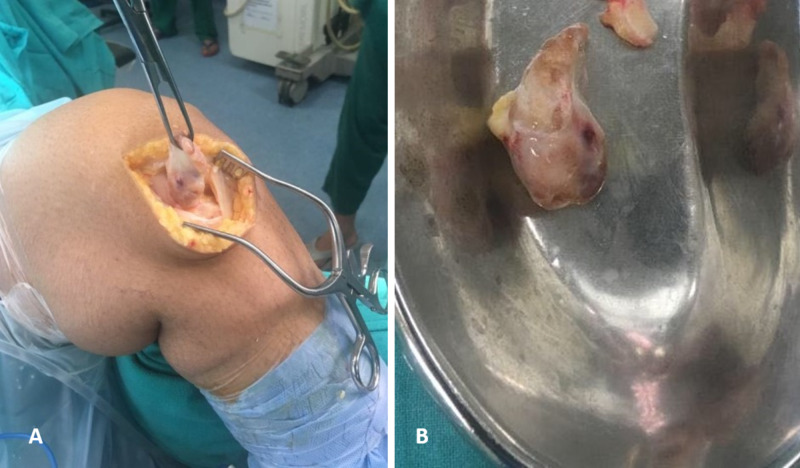
Intraoperative findings. (A) Surgical approach - medial parapatellar. (B) Completely excised mass - grayish yellow with a thin capsule.

**Figure 4:**
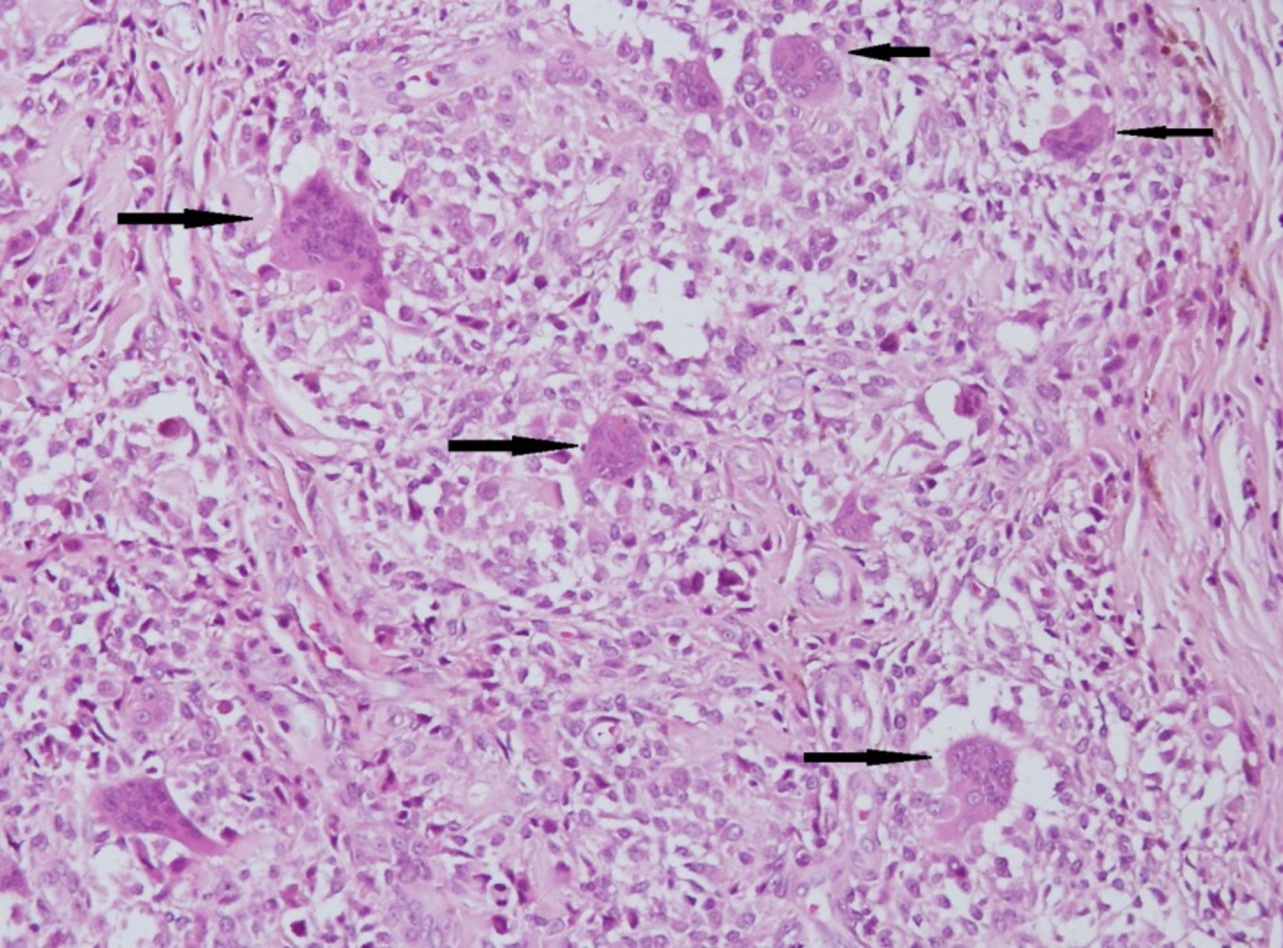
Histopathological section of the specimen showing multinucleated giant cells (black arrows) in a stroma of mononuclear cells (hematoxylin and eosin stain, × 100).

Due to the size of the lesion and its location near the anteromedial portal site (posing increased risk of perforation during insertion of arthroscopic instruments), open resection of the mass was planned. A left knee joint arthrotomy using a medial parapatellar approach was performed (Figure 3A). Swelling was present within Hoffa's fat pad and removed en masse (Figure 3B). Grossly, the mass was a single nodular circumscribed thinly encapsulated piece of tissue measuring approximately 5 × 3 × 3 cm. The outer surface was focally fat-laden, and the cut surface was gray-brown to gray-yellow. Microscopically, it was composed of mononuclear stromal cells, osteoclastic giant cells, and hyalinized stroma with few lymphocytes and macrophages, which confirmed the diagnosis of TGCT (Figure 4).

The postoperative period was uneventful. Sutures were removed on the 10th postoperative day, and there was regular follow-up. The Oxford knee score had increased to 52 at three months and 56 at six months post-surgery. Follow-up at 20 months indicated that the patient was asymptomatic with an Oxford knee score of 58 and had near full range of motion in the affected knee. She was able to return to her previous level of daily activities, and there was no local recurrence of the tumor.

## Discussion

The localized form and diffuse form of giant cell tumors have been classified by the World Health Organization as fibrocystic tumors [6]. TGCT mainly involves the digits of the hand and foot, whereas the diffuse form manifests as extensive involvement of the synovium and capsule [2,3].

The presence of TGCT in Hoffa's fat pad is a very rare finding [4]. TGCT is usually seen in the age group of 30 to 50 years and more commonly in women, and our patient was a 33-year-old woman [1].

Due to nonspecific signs and symptoms, diagnosis of a giant cell tumor can be delayed for weeks or months. The common concerns may include painless or painful palpable swelling, joint effusion, locking of the knee joint, and restriction of movement [1,7,8].

The size of the tumor in this patient was 5 cm at its largest dimension. Only a few cases of a large tumor of this size (in the infrapatellar region) have been reported in the literature [1,3,7,9]. Panagopoulos et al. reported a case of a giant cell tumor measuring 5 cm [8]. Radiographs are of little help in diagnosing giant cell tumors because they are normal for a majority of the time, but sometimes an opacity may be observed in the infrapatellar region [1,2,8,9].

Being uncommon in the knee, the unusual presence of TGCT can mimic other swellings in Hoffa's fat pad, such as ganglion cyst, hemangioma, lipoma, focal villonodular synovitis, synovial chondromatosis, or osteochondroma. MRI is an important tool that assists in diagnosing these lesions [10].

Findings specific for giant cell tumors show isointensity to muscle in T1-weighted images, whereas T2 images show low to intermediate signal intensity and intense homogeneous enhancement after contrast administration [1,7]. The diagnosis of giant cell tumors is made on a histopathological basis, which shows multinucleated giant cells with round or polygonal stromal cells [1,2,8].

Complete excision of the tumor is the treatment of choice. Depending upon the site, size, and extent of the lesion, the tumor mass can be resected either by open excision, arthroscopic procedure, or arthroscopic-assisted open excision [2,7]. A review of different cases is summarized in Table 1.

**Table 1:** Review of different cases with their clinical features, treatment methods, and follow-up. N/A, not available.

Author	Number of Cases Reported	Age	Sex	Clinical Concern	Size	Treatment	Follow-up	Recurrence
Kılıçaslan et al. [1]	1	32 years	Female	Anterior knee pain and swelling	3.5 × 3 × 1.8 cm	Open excision	18 months	No
Kim et al. [4]	1	45 years	Female	Extension limitation and pain	2.5 × 3 × 4 cm	Arthroscopic excision	12 months	No
Chechik et al [7]	2	48 years	Female	Nontender palpable swelling	2 × 2 × 2.5 cm	Arthroscopic assisted open excision	36 months	No
		37 years	Female	Swelling with intermittent locking	N/A	Arthroscopic assisted open excision	24 months	No
Panagopoulos et al. [8]	1	26 years	Male	Painful locked knee	5 × 4 × 2 cm	Open excision	24 months	No
Abdullah et al. [9]	1	11 years	Female	Painful lump	3 × 3.5 × 1.5 cm	Open excision	35 months	No
Present study	1	33 years	Female	Swelling and pain	5 × 3 × 3 cm	Open excision	20 months	No

The presence of residual tissue is the main cause of recurrence. We proceeded with the removal by performing open resection of the tumor due to its large size and also because the recurrence rate is less after open resection, as it allows complete removal of the tumor mass [1,2,8,9].

## Conclusions

Although TGCT is a rare occurrence in Hoffa's fat pad, it can be a cause of nontraumatic knee swelling and pain. Management can be delayed if an early diagnosis is not made. Hence, while making a differential diagnosis for symptoms of nontraumatic knee swelling and pain, TGCT should be considered as a potential cause. Proper knowledge of this disorder can assist in timely intervention to prevent morbidity to the patient.
